# Ethyl 2-[2-(4-hy­droxy-3-meth­oxy­benzyl­idene)hydrazin-1-yl­idene]-3,4-dimethyl-2,3-dihydro-1,3-thia­zole-5-carboxyl­ate

**DOI:** 10.1107/S1600536812043772

**Published:** 2012-10-27

**Authors:** Sema Öztürk Yildirim, Ray J. Butcher, Yavuz Köysal, Esen Nur Kantar, Ayşe Belder

**Affiliations:** a Department of Chemistry, Howard University, 525 College Street NW, Washington, DC 20059, USA; b Department of Physics, Faculty of Sciences, Erciyes University, 38039 Kayseri, Turkey; c Yesilyurt Demir Celik Vocational School, Ondokuz Mayis University, Samsun, Turkey; d Department of Physics, Faculty of Arts and Sciences, Ondokuz Mayis University, TR-55139 Samsun, Turkey; e Department of Chemistry, Karabük University, 78200 Karabük, Turkey

## Abstract

The title compound, C_16_H_19_N_3_O_4_S, is almost planar, with a dihedral angle of 2.88 (9)° between the mean planes of the benzene and thia­zole rings. The mol­ecule adopts an *E* conformation about the two C=N bonds, with a C—N—N—C torsion angle of −177.01 (11)°. An intra­molecular C—H⋯O hydrogen bond exists between a thia­zole methyl group and the formic acid ethyl ester carbonyl O atom. In the crystal, mol­ecules are linked by O—-H⋯O hydrogen bonds, forming chains propagating along [2-10]. The chains are linked *via* C—H⋯O hydrogen bonds with *R*_2_^2^(12) ring motifs, forming sheets lying parallel to (12-2). The sheets are further linked through out-of-plane C—H⋯N hydrogen bonds with *R*_2_^2^(12) ring motifs and C—H⋯π inter­actions, forming an inter­esting three-dimensional supra­molecular architecture.

## Related literature

For the various biological activities of 1,3-thia­zoles, 1,3,4-thia­diazo­les and their derivatives, see: Shucla *et al.* (1984 ▶); Desai & Baxi (1992 ▶); Mullican *et al.* (1993 ▶); Chapleo *et al.* (1986 ▶); Turner *et al.* (1988 ▶); Mazzone *et al.* (1993 ▶); Miyamoto *et al.* (1985 ▶); Hanna *et al.* (1995 ▶); Oh *et al.* (2002 ▶). For the anti­microbial activity of thia­diazo­les and related compounds, see: Sancak *et al.* (2007 ▶). For bond lengths of structurally related mol­ecules, see: Imhof & Wunderle (2012 ▶); Randell *et al.* (2012 ▶). For details of the Cambridge Structural Database, see: Allen (2002 ▶). For synthetic details, see: Er *et al.* (2009 ▶). For graph-set notation, see: Bernstein *et al.* (1995 ▶).
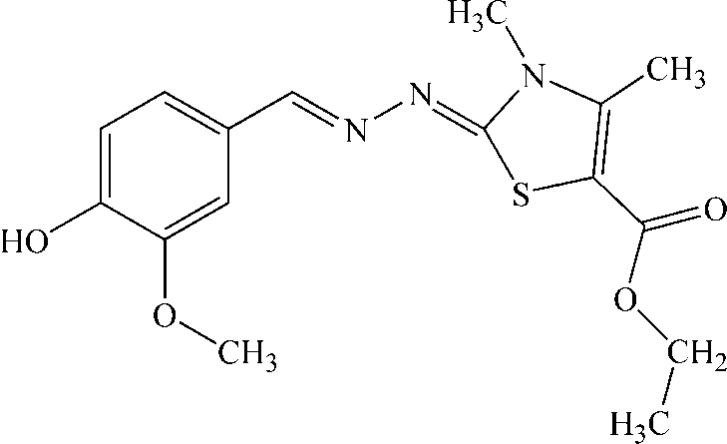


## Experimental

### 

#### Crystal data

C_16_H_19_N_3_O_4_S
*M* *_r_* = 349.40
Triclinic, 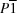

*a* = 6.8957 (3) Å
*b* = 10.2716 (5) Å
*c* = 12.7297 (6) Å
α = 74.843 (4)°
β = 87.579 (4)°
γ = 73.304 (4)°
*V* = 833.06 (7) Å^3^
*Z* = 2
Cu *K*α radiation
μ = 1.96 mm^−1^
*T* = 123 K
0.40 × 0.35 × 0.30 mm

#### Data collection

Agilent Xcalibur (Ruby, Gemini) diffractometer
Absorption correction: multi-scan (*CrysAlis RED*; Agilent, 2011 ▶) *T*_min_ = 0.508, *T*_max_ = 0.591
5272 measured reflections
3316 independent reflections
3254 reflections with *I* > 2σ(*I*)
*R*_int_ = 0.016

#### Refinement

*R*[*F* ^2^ > 2σ(*F* ^2^)] = 0.036
*wR*(*F* ^2^) = 0.099
*S* = 1.02
3316 reflections
222 parameters
H-atom parameters constrained
Δρ_max_ = 0.35 e Å^−3^
Δρ_min_ = −0.29 e Å^−3^

### 

Data collection: *CrysAlis PRO* (Agilent, 2011 ▶); cell refinement: *CrysAlis PRO*; data reduction: *CrysAlis PRO*; program(s) used to solve structure: *SHELXS97* (Sheldrick, 2008 ▶); program(s) used to refine structure: *SHELXL97* (Sheldrick, 2008 ▶); molecular graphics: *SHELXTL* (Sheldrick, 2008 ▶); software used to prepare material for publication: *SHELXTL*.

## Supplementary Material

Crystal structure: contains datablock(s) I, global. DOI: 10.1107/S1600536812043772/su2516sup1.cif

Structure factors: contains datablock(s) I. DOI: 10.1107/S1600536812043772/su2516Isup2.hkl

Supplementary material file. DOI: 10.1107/S1600536812043772/su2516Isup3.cml

Additional supplementary materials:  crystallographic information; 3D view; checkCIF report

## Figures and Tables

**Table 1:** Hydrogen-bond geometry (Å, °) *Cg*1 is the centroid of the C10–C15 ring.

*D*—H⋯*A*	*D*—H	H⋯*A*	*D*⋯*A*	*D*—H⋯*A*
C7—H7*A*⋯O2	0.98	2.28	3.0111 (19)	130
O4—H4*O*⋯O2^i^	0.84	1.85	2.6878 (14)	176
C16—H16*A*⋯O4^ii^	0.98	2.41	3.3824 (18)	171
C8—H8*C*⋯N3^iii^	0.98	2.62	3.3986 (19)	137
C6—H6*B*⋯*Cg*1^iv^	0.98	2.96	3.6414 (16)	128
C7—H7*C*⋯*Cg*1^v^	0.98	2.62	3.4762 (16)	146

Symmetry codes: (i) 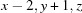; (ii) 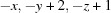; (iii) 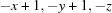; (iv) 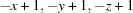; (v) 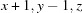.

**Fig. 1.**
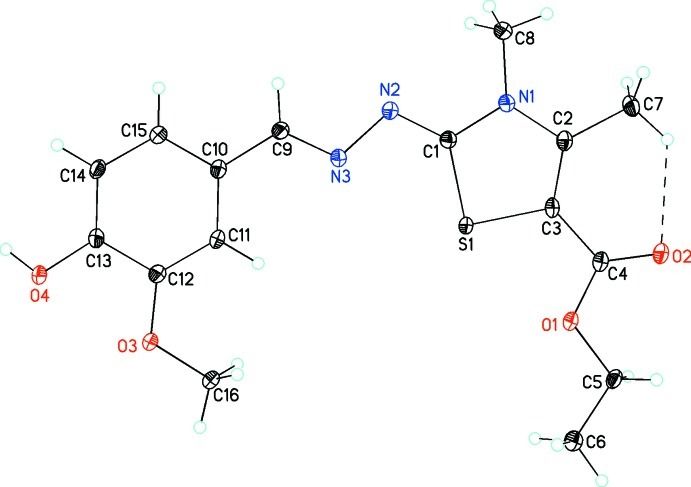
The molecular structure of the title molecule with the atom numbering. The displacement ellipsoids are drawn at the 30% probability level. The intramolecular C—H···O hydrogen bond is shown as a dashed line.

**Fig. 2.**
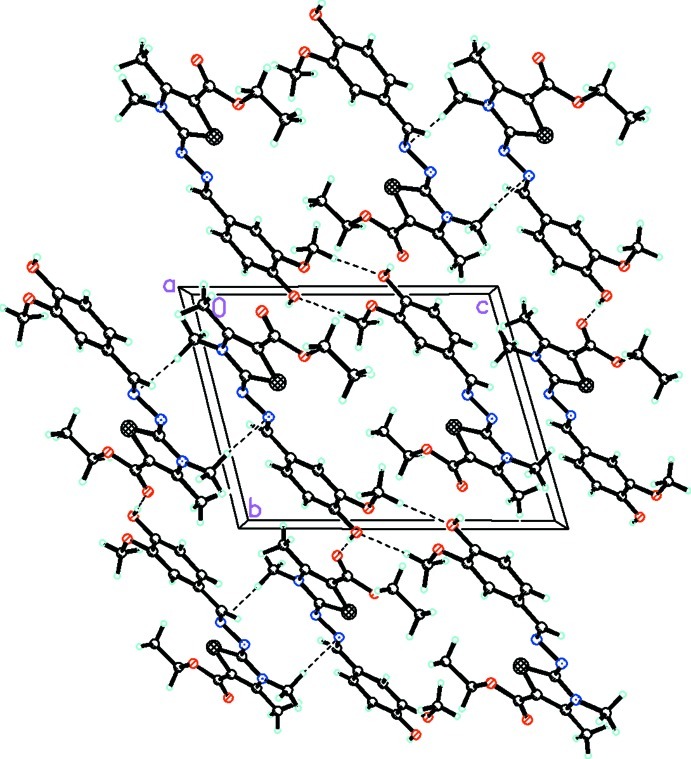
The crystal packing of the title compound, viewed along the a axis. Hydrogen bonds are shown as dashed lines - see Table 1 for details.

## supplementary crystallographic information

### Comment

1,3-thiazoles, 1,3,4-thiadiazoles and their derivatives exhibit various biological activities, such as antituberculosis (Shucla,*et al.*, 1984), antimicrobial (Desai & Baxi, 1992), anti-inflammatory (Mullican *et al.*, 1993), antiviral, anticonvulsant (Chapleo *et al.*, 1986), antihypertensive (Turner *et al.*, 1988), local anesthetic (Mazzone *et al.*, 1993), anticancer (Miyamoto *et al.*, 1985), hypoglycemic (Hanna *et al.*, 1995), and cytotoxic activities (Oh *et al.*, 2002). Thiadiazoles and related compounds are of great interest in chemistry owing to their bioactivity with certain plant growth regulating effects as well as antimicrobial activity (Sancak *et al.*, 2007). Owing to the importance of these 1,3,4-thiadiazoles derivatives, we report herein on the crystal structure of the title compound.

The molecular structure of the title compound is shown in Fig. 1. The N2—N3 single bond [1.3982 (16) Å] and the N2═C1 double bond [1.2958 (18) Å] distances are in the normal range and are comparable with those found for similar compounds (Imhof & Wunderle, 2012; Randell *et al.*, 2012). Bond lengths and angles can be regarded as normal (Allen, 2002). The molecule adopts an *E* conformation about the C1═N2 and the C9═N3 bonds with a C9—N3—N2—C1 torsion angle of -177.01 (11) °. The 2-methoxy-phenol ring (C10—C15) and the thiazole ring (C1/N1/C2/C3/S1) are coplanar with a dihedral angle between their mean planes of only 2.88 (9) °. An intramolecular C—H···O hydrogen bond exists between the thiazol methyl group and atom O2 of the formic acid ethyl ester C═O O atom.

In the crystal, an interesting supramolecular architecture is formed as the molecules link up to form sheets in plane (1 2 -2) through both C—H···O *R*^2^_2_(12) ring motifs (Bernstein *et al.*, 1995) and O—H···O interactions. These sheets are further linked through out-of-plane C—H···N *R*^2^_2_(12) ring motifs and C-H···π interactions (Table 1 and Fig. 2).

### Experimental

The title compound was synthesized according to the published procedure (Er *et al.*, 2009). Crystals were grown by slow evaporation of a 1 3-dichloro-2-propanol solution.

### Refinement

The H atoms were placed in calculated positions and refined in the riding mode: O—H = 0.84 Å, C—H = 0.95–0.98 Å with *U*_iso_(H) = 1.5*U*_eq_(C) for methyl H atoms and = 1.2*U*_eq_(O,C) for other H atoms.

### Crystal data

**Table:**

C_16_H_19_N_3_O_4_S	*Z* = 2
*M**_r_* = 349.40	*F*(000) = 368
Triclinic, *P*1	*D*_x_ = 1.393 Mg m^−^^3^
Hall symbol: -P 1	Cu *K*α radiation, λ = 1.54184 Å
*a* = 6.8957 (3) Å	Cell parameters from 4259 reflections
*b* = 10.2716 (5) Å	θ = 3.6–75.0°
*c* = 12.7297 (6) Å	µ = 1.96 mm^−^^1^
α = 74.843 (4)°	*T* = 123 K
β = 87.579 (4)°	Block, yellow
γ = 73.304 (4)°	0.40 × 0.35 × 0.30 mm
*V* = 833.06 (7) Å^3^	

### Data collection

**Table:**

Agilent Xcalibur (Ruby, Gemini) diffractometer	3316 independent reflections
Radiation source: Enhance (Cu) X-ray Source	3254 reflections with *I* > 2σ(*I*)
Graphite monochromator	*R*_int_ = 0.016
Detector resolution: 10.5081 pixels mm^-1^	θ_max_ = 75.2°, θ_min_ = 3.6°
ω scans	*h* = −8→6
Absorption correction: multi-scan (*CrysAlis RED*; Agilent, 2011)	*k* = −12→12
*T*_min_ = 0.508, *T*_max_ = 0.591	*l* = −14→15
5272 measured reflections	

### Refinement

**Table:**

Refinement on *F*^2^	Primary atom site location: structure-invariant direct methods
Least-squares matrix: full	Secondary atom site location: difference Fourier map
*R*[*F*^2^ > 2σ(*F*^2^)] = 0.036	Hydrogen site location: inferred from neighbouring sites
*wR*(*F*^2^) = 0.099	H-atom parameters constrained
*S* = 1.02	*w* = 1/[σ^2^(*F*_o_^2^) + (0.0628*P*)^2^ + 0.3221*P*] where *P* = (*F*_o_^2^ + 2*F*_c_^2^)/3
3316 reflections	(Δ/σ)_max_ < 0.001
222 parameters	Δρ_max_ = 0.35 e Å^−^^3^
0 restraints	Δρ_min_ = −0.29 e Å^−^^3^

### Special details

**Table:**

Geometry. All e.s.d.'s (except the e.s.d. in the dihedral angle between two l.s. planes) are estimated using the full covariance matrix. The cell e.s.d.'s are taken into account individually in the estimation of e.s.d.'s in distances, angles and torsion angles; correlations between e.s.d.'s in cell parameters are only used when they are defined by crystal symmetry. An approximate (isotropic) treatment of cell e.s.d.'s is used for estimating e.s.d.'s involving l.s. planes.
Refinement. Refinement of *F*^2^ against ALL reflections. The weighted *R*-factor *wR* and goodness of fit *S* are based on *F*^2^, conventional *R*-factors *R* are based on *F*, with *F* set to zero for negative *F*^2^. The threshold expression of *F*^2^ > σ(*F*^2^) is used only for calculating *R*-factors(gt) *etc*. and is not relevant to the choice of reflections for refinement. *R*-factors based on *F*^2^ are statistically about twice as large as those based on *F*, and *R*- factors based on ALL data will be even larger.

### Fractional atomic coordinates and isotropic or equivalent isotropic displacement parameters (Å^2^)

**Table:**

	*x*	*y*	*z*	*U*_iso_*/*U*_eq_	
S1	0.70492 (5)	0.40814 (3)	0.24740 (3)	0.02041 (12)	
N1	0.66196 (18)	0.27633 (12)	0.10704 (9)	0.0211 (3)	
N2	0.36294 (18)	0.44474 (12)	0.12687 (9)	0.0215 (2)	
N3	0.28500 (18)	0.54591 (12)	0.18464 (9)	0.0209 (2)	
O1	1.09737 (14)	0.30755 (10)	0.35164 (8)	0.0228 (2)	
O2	1.25414 (15)	0.14195 (11)	0.26529 (9)	0.0262 (2)	
O3	0.01284 (15)	0.91719 (11)	0.41058 (8)	0.0250 (2)	
O4	−0.36639 (15)	1.02884 (11)	0.35124 (9)	0.0260 (2)	
H4O	−0.4861	1.0602	0.3258	0.031*	
C1	0.5524 (2)	0.38117 (14)	0.15329 (11)	0.0197 (3)	
C2	0.8616 (2)	0.21847 (14)	0.14284 (11)	0.0209 (3)	
C3	0.9110 (2)	0.27517 (14)	0.21950 (11)	0.0207 (3)	
C4	1.1041 (2)	0.23365 (14)	0.27913 (11)	0.0209 (3)	
C5	1.2838 (2)	0.27620 (15)	0.41589 (11)	0.0229 (3)	
H5A	1.3993	0.2794	0.3674	0.027*	
H5B	1.3131	0.1810	0.4664	0.027*	
C6	1.2533 (2)	0.38483 (16)	0.47858 (12)	0.0277 (3)	
H6A	1.3767	0.3671	0.5220	0.042*	
H6B	1.1399	0.3799	0.5270	0.042*	
H6C	1.2234	0.4786	0.4278	0.042*	
C7	0.9959 (2)	0.10729 (15)	0.09540 (12)	0.0256 (3)	
H7A	1.1327	0.0781	0.1283	0.038*	
H7B	1.0011	0.1449	0.0165	0.038*	
H7C	0.9421	0.0260	0.1102	0.038*	
C8	0.5613 (2)	0.23358 (15)	0.02873 (12)	0.0250 (3)	
H8A	0.4387	0.2114	0.0602	0.037*	
H8B	0.6532	0.1502	0.0113	0.037*	
H8C	0.5243	0.3105	−0.0379	0.037*	
C9	0.0948 (2)	0.60606 (14)	0.16368 (11)	0.0211 (3)	
H9A	0.0275	0.5778	0.1138	0.025*	
C10	−0.0221 (2)	0.71595 (14)	0.21327 (11)	0.0205 (3)	
C11	0.0644 (2)	0.76348 (14)	0.28849 (11)	0.0199 (3)	
H11A	0.2047	0.7247	0.3077	0.024*	
C12	−0.0528 (2)	0.86614 (14)	0.33475 (11)	0.0206 (3)	
C13	−0.2607 (2)	0.92734 (14)	0.30389 (11)	0.0208 (3)	
C14	−0.3465 (2)	0.88063 (15)	0.22958 (11)	0.0228 (3)	
H14A	−0.4862	0.9207	0.2091	0.027*	
C15	−0.2279 (2)	0.77488 (15)	0.18495 (11)	0.0231 (3)	
H15A	−0.2880	0.7426	0.1347	0.028*	
C16	0.2148 (2)	0.84763 (16)	0.45449 (12)	0.0271 (3)	
H16A	0.2424	0.8898	0.5108	0.041*	
H16B	0.3111	0.8580	0.3963	0.041*	
H16C	0.2290	0.7475	0.4864	0.041*	

### Atomic displacement parameters (Å^2^)

**Table:**

	*U*^11^	*U*^22^	*U*^33^	*U*^12^	*U*^13^	*U*^23^
S1	0.01519 (18)	0.02052 (19)	0.02471 (19)	−0.00053 (13)	−0.00105 (12)	−0.00933 (13)
N1	0.0198 (6)	0.0202 (6)	0.0223 (5)	−0.0022 (5)	−0.0005 (4)	−0.0073 (4)
N2	0.0189 (6)	0.0207 (6)	0.0236 (6)	−0.0022 (4)	−0.0011 (4)	−0.0073 (4)
N3	0.0190 (6)	0.0203 (6)	0.0219 (5)	−0.0023 (5)	0.0000 (4)	−0.0068 (4)
O1	0.0151 (5)	0.0248 (5)	0.0263 (5)	−0.0009 (4)	−0.0017 (4)	−0.0079 (4)
O2	0.0169 (5)	0.0255 (5)	0.0330 (5)	0.0010 (4)	−0.0016 (4)	−0.0095 (4)
O3	0.0170 (5)	0.0275 (5)	0.0296 (5)	0.0007 (4)	−0.0047 (4)	−0.0131 (4)
O4	0.0167 (5)	0.0278 (5)	0.0314 (5)	0.0023 (4)	−0.0019 (4)	−0.0135 (4)
C1	0.0195 (7)	0.0189 (6)	0.0201 (6)	−0.0043 (5)	0.0003 (5)	−0.0052 (5)
C2	0.0188 (6)	0.0182 (6)	0.0226 (6)	−0.0026 (5)	0.0023 (5)	−0.0030 (5)
C3	0.0160 (6)	0.0184 (6)	0.0247 (6)	−0.0005 (5)	0.0024 (5)	−0.0053 (5)
C4	0.0167 (6)	0.0191 (6)	0.0239 (6)	−0.0030 (5)	0.0017 (5)	−0.0027 (5)
C5	0.0150 (6)	0.0254 (7)	0.0250 (7)	−0.0018 (5)	−0.0026 (5)	−0.0049 (5)
C6	0.0235 (7)	0.0273 (7)	0.0301 (7)	−0.0027 (6)	−0.0025 (6)	−0.0080 (6)
C7	0.0248 (7)	0.0225 (7)	0.0268 (7)	−0.0009 (6)	0.0038 (6)	−0.0087 (5)
C8	0.0240 (7)	0.0251 (7)	0.0264 (7)	−0.0039 (6)	−0.0019 (6)	−0.0110 (6)
C9	0.0200 (7)	0.0211 (6)	0.0213 (6)	−0.0048 (5)	−0.0014 (5)	−0.0049 (5)
C10	0.0181 (7)	0.0196 (6)	0.0210 (6)	−0.0028 (5)	0.0003 (5)	−0.0033 (5)
C11	0.0143 (6)	0.0200 (6)	0.0219 (6)	−0.0019 (5)	−0.0008 (5)	−0.0027 (5)
C12	0.0192 (7)	0.0213 (6)	0.0197 (6)	−0.0049 (5)	−0.0004 (5)	−0.0037 (5)
C13	0.0176 (6)	0.0201 (6)	0.0215 (6)	−0.0019 (5)	0.0017 (5)	−0.0041 (5)
C14	0.0146 (6)	0.0251 (7)	0.0255 (7)	−0.0012 (5)	−0.0022 (5)	−0.0055 (5)
C15	0.0195 (7)	0.0248 (7)	0.0244 (7)	−0.0038 (5)	−0.0025 (5)	−0.0075 (5)
C16	0.0189 (7)	0.0310 (7)	0.0303 (7)	−0.0010 (6)	−0.0062 (6)	−0.0121 (6)

### Geometric parameters (Å, º)

**Table:**

S1—C1	1.7507 (14)	C6—H6B	0.9800
S1—C3	1.7626 (14)	C6—H6C	0.9800
N1—C2	1.3773 (18)	C7—H7A	0.9800
N1—C1	1.3795 (18)	C7—H7B	0.9800
N1—C8	1.4599 (17)	C7—H7C	0.9800
N2—C1	1.2958 (18)	C8—H8A	0.9800
N2—N3	1.3982 (16)	C8—H8B	0.9800
N3—C9	1.2847 (18)	C8—H8C	0.9800
O1—C4	1.3315 (17)	C9—C10	1.4592 (19)
O1—C5	1.4624 (16)	C9—H9A	0.9500
O2—C4	1.2247 (17)	C10—C15	1.3951 (19)
O3—C12	1.3601 (17)	C10—C11	1.4039 (19)
O3—C16	1.4307 (16)	C11—C12	1.3808 (19)
O4—C13	1.3551 (17)	C11—H11A	0.9500
O4—H4O	0.8400	C12—C13	1.4159 (19)
C2—C3	1.359 (2)	C13—C14	1.387 (2)
C2—C7	1.4941 (19)	C14—C15	1.394 (2)
C3—C4	1.457 (2)	C14—H14A	0.9500
C5—C6	1.498 (2)	C15—H15A	0.9500
C5—H5A	0.9900	C16—H16A	0.9800
C5—H5B	0.9900	C16—H16B	0.9800
C6—H6A	0.9800	C16—H16C	0.9800
			
C1—S1—C3	89.88 (6)	C2—C7—H7C	109.5
C2—N1—C1	114.90 (11)	H7A—C7—H7C	109.5
C2—N1—C8	125.69 (12)	H7B—C7—H7C	109.5
C1—N1—C8	119.40 (11)	N1—C8—H8A	109.5
C1—N2—N3	110.82 (11)	N1—C8—H8B	109.5
C9—N3—N2	112.08 (11)	H8A—C8—H8B	109.5
C4—O1—C5	116.48 (11)	N1—C8—H8C	109.5
C12—O3—C16	116.76 (11)	H8A—C8—H8C	109.5
C13—O4—H4O	109.5	H8B—C8—H8C	109.5
N2—C1—N1	121.36 (12)	N3—C9—C10	122.79 (13)
N2—C1—S1	128.22 (11)	N3—C9—H9A	118.6
N1—C1—S1	110.42 (10)	C10—C9—H9A	118.6
C3—C2—N1	112.65 (12)	C15—C10—C11	119.09 (13)
C3—C2—C7	127.97 (13)	C15—C10—C9	118.38 (12)
N1—C2—C7	119.37 (12)	C11—C10—C9	122.53 (12)
C2—C3—C4	127.31 (12)	C12—C11—C10	120.55 (12)
C2—C3—S1	112.14 (10)	C12—C11—H11A	119.7
C4—C3—S1	120.50 (11)	C10—C11—H11A	119.7
O2—C4—O1	123.89 (13)	O3—C12—C11	125.70 (13)
O2—C4—C3	124.66 (13)	O3—C12—C13	114.35 (12)
O1—C4—C3	111.45 (11)	C11—C12—C13	119.95 (13)
O1—C5—C6	107.59 (11)	O4—C13—C14	123.43 (13)
O1—C5—H5A	110.2	O4—C13—C12	116.96 (12)
C6—C5—H5A	110.2	C14—C13—C12	119.61 (13)
O1—C5—H5B	110.2	C13—C14—C15	120.03 (13)
C6—C5—H5B	110.2	C13—C14—H14A	120.0
H5A—C5—H5B	108.5	C15—C14—H14A	120.0
C5—C6—H6A	109.5	C14—C15—C10	120.74 (13)
C5—C6—H6B	109.5	C14—C15—H15A	119.6
H6A—C6—H6B	109.5	C10—C15—H15A	119.6
C5—C6—H6C	109.5	O3—C16—H16A	109.5
H6A—C6—H6C	109.5	O3—C16—H16B	109.5
H6B—C6—H6C	109.5	H16A—C16—H16B	109.5
C2—C7—H7A	109.5	O3—C16—H16C	109.5
C2—C7—H7B	109.5	H16A—C16—H16C	109.5
H7A—C7—H7B	109.5	H16B—C16—H16C	109.5
			
C1—N2—N3—C9	−177.01 (11)	S1—C3—C4—O2	179.55 (11)
N3—N2—C1—N1	179.73 (11)	C2—C3—C4—O1	−177.28 (13)
N3—N2—C1—S1	0.40 (17)	S1—C3—C4—O1	−0.06 (16)
C2—N1—C1—N2	179.91 (12)	C4—O1—C5—C6	171.31 (11)
C8—N1—C1—N2	−1.54 (19)	N2—N3—C9—C10	−179.74 (11)
C2—N1—C1—S1	−0.65 (15)	N3—C9—C10—C15	179.88 (13)
C8—N1—C1—S1	177.90 (10)	N3—C9—C10—C11	−0.5 (2)
C3—S1—C1—N2	179.35 (13)	C15—C10—C11—C12	0.6 (2)
C3—S1—C1—N1	−0.04 (10)	C9—C10—C11—C12	−178.93 (12)
C1—N1—C2—C3	1.22 (17)	C16—O3—C12—C11	−7.2 (2)
C8—N1—C2—C3	−177.22 (12)	C16—O3—C12—C13	172.68 (12)
C1—N1—C2—C7	−178.01 (11)	C10—C11—C12—O3	177.97 (12)
C8—N1—C2—C7	3.6 (2)	C10—C11—C12—C13	−1.9 (2)
N1—C2—C3—C4	176.20 (13)	O3—C12—C13—O4	1.00 (18)
C7—C2—C3—C4	−4.7 (2)	C11—C12—C13—O4	−179.11 (12)
N1—C2—C3—S1	−1.21 (15)	O3—C12—C13—C14	−178.01 (12)
C7—C2—C3—S1	177.93 (11)	C11—C12—C13—C14	1.9 (2)
C1—S1—C3—C2	0.71 (11)	O4—C13—C14—C15	−179.53 (13)
C1—S1—C3—C4	−176.90 (11)	C12—C13—C14—C15	−0.6 (2)
C5—O1—C4—O2	1.04 (19)	C13—C14—C15—C10	−0.7 (2)
C5—O1—C4—C3	−179.34 (11)	C11—C10—C15—C14	0.7 (2)
C2—C3—C4—O2	2.3 (2)	C9—C10—C15—C14	−179.74 (12)

### Hydrogen-bond geometry (Å, º)

Cg1 is the centroid of the C10–C15 ring.

**Table:**

*D*—H···*A*	*D*—H	H···*A*	*D*···*A*	*D*—H···*A*
C7—H7*A*···O2	0.98	2.28	3.0111 (19)	130
O4—H4*O*···O2^i^	0.84	1.85	2.6878 (14)	176
C16—H16*A*···O4^ii^	0.98	2.41	3.3824 (18)	171
C8—H8*C*···N3^iii^	0.98	2.62	3.3986 (19)	137
C6—H6*B*···*Cg*1^iv^	0.98	2.96	3.6414 (16)	128
C7—H7*C*···*Cg*1^v^	0.98	2.62	3.4762 (16)	146

Symmetry codes: (i) *x*−2, *y*+1, *z*; (ii) −*x*, −*y*+2, −*z*+1; (iii) −*x*+1, −*y*+1, −*z*; (iv) −*x*+1, −*y*+1, −*z*+1; (v) *x*+1, *y*−1, *z*.
